# Piece by piece—a computer-aided method for virtual re-association of commingled fragmented remains

**DOI:** 10.1093/fsr/owae035

**Published:** 2024-07-15

**Authors:** Lise Malfroy Camine, Virginie Magnin, Ruben Soto, Christine Bruguier, Silke Grabherr, Vincent Varlet, Negahnaz Moghaddam

**Affiliations:** University Center of Legal Medicine Lausanne-Geneva, Geneva University Hospitals and University of Geneva, Lausanne, Switzerland; Swiss Human Institute of Forensic Taphonomy, University Center of Legal Medicine Lausanne-Geneva, Lausanne, Switzerland; Forensic Sciences Institute of the French Gendarmerie, Institut de Recherche Criminelle de la Gendarmerie Nationale (IRCGN), Pontoise, France; Forensic Imaging and Anthropology Unit, University Center of Legal Medicine Lausanne-Geneva, Lausanne, Switzerland; Forensic Imaging and Anthropology Unit, University Center of Legal Medicine Lausanne-Geneva, Lausanne, Switzerland; Forensic Imaging and Anthropology Unit, University Center of Legal Medicine Lausanne-Geneva, Lausanne, Switzerland; Department of Diagnostic and Interventional Radiology, Lausanne University Hospital, Lausanne, Switzerland; University Center of Legal Medicine Lausanne-Geneva, Geneva University Hospitals and University of Geneva, Lausanne, Switzerland; Swiss Human Institute of Forensic Taphonomy, University Center of Legal Medicine Lausanne-Geneva, Lausanne, Switzerland; Swiss Human Institute of Forensic Taphonomy, University Center of Legal Medicine Lausanne-Geneva, Lausanne, Switzerland; Forensic Imaging and Anthropology Unit, University Center of Legal Medicine Lausanne-Geneva, Lausanne, Switzerland; Forensic Pathology Unit, University Center of Legal Medicine Lausanne-Geneva, Lausanne, Switzerland

**Keywords:** forensic anthropology, fragmented human remains, disaster victim identification, 3D, virtual re-association

## Abstract

Air crashes or explosions with numerous victims may result in thousands of fragmented human remains that present a massive challenge for disaster victim identification teams. Genetic identification may present important financial and technical limits, and the physical re-association of fractured bones by forensic anthropologists may require a time-consuming phase of cleaning and drying. A virtual re-association (VRA) of fragmented human remains using postmortem computed tomography (CT)-scan images could enhance the identification process and reduce the number of genetic analyses required. Therefore, this study investigated the advantages and limitations of a VRA protocol in comparison with physical re-association (PRA) in a laboratory setting and in a real case scenario. As a first step, six porcine femurs were scanned by multi-detector CT before and after physical fragmentation. PRA of the dry bones and VRA of the 3D models of the fragments were then performed. The physically reconstructed dry bones were then once more scanned with CT. The mean distance between intact and reconstructed models, the number of re-associated fragments, and the time needed for the reconstruction were evaluated. In a second step, 87 fragmented remains resulting from a controlled pig bombing were collected, scanned, and virtually re-associated to test the feasibility of the protocol in a real context. The reconstruction of the femurs showed no difference in accuracy between PRA and VRA. Although the VRA was faster than PRA, the preparation of the material still needs to be taken into consideration. The VRA after the controlled pig bombing was limited to 8% of the total fragments. Differences in alveolar and cortical osseous structure and the presence of cartilage resulted in segmentation approximations and difficulties in the re-association itself. The explosion produced an important loss of intermediate bone elements. The VRA method still needs further evaluations with a larger sample size and different fragmentation mechanisms. However, the presented research shows promising results towards enhancing the efficiency of identifying individuals after a mass disaster.

**Key points:**

- Mass disasters with highly fragmented bodies represent a major challenge for the disaster victim identification (DVI) teams.
- Using CT-scans of the fragments, a virtual re-association method could enhance the DVI process and reduce the number of genetic analysis required.
- A laboratory setting using porcine femurs shown that the VRA process could start earlier and was faster than the physical re-association.
- Although methodological limits were highlighted, and the efficiency of the method has to be re-evaluated, the controlled bombing showed that the VRA could be applied in a real case scenario.

## Introduction

In situations faced with numerous fragmented human remains (FHRs), the forensic teams involved face the challenging and tedious task of re-associating all the recovered human remains with the corresponding identities of the people involved. This identification step is a prerequisite for the grieving process of the victims’ families, for the recognition of the death of their beloved ones, and for legal proceedings [1]. However, some situations such as air crashes, explosions, or intensive fires may result in the bodies suffering a high degree of fragmentation, and thus to even more complex disaster victim identification (DVI) missions [2, 3]. As an example, after the World Trade Center attack, the recovery units collected 21 906 human fragments, of which 33% still remained unidentified in 2019 [2, 4], despite ongoing multidisciplinary identification procedures, including an intensive DNA sampling and analysis process.

During the DVI process, genetic, forensic odontology, and fingerprint analyses are the three major identification methods investigated for each body element and missing person, and are sufficient by themselves to assess a formal match [5, 6]. Secondary identifiers such as property (e.g. clothes or jewels) or medical features (e.g. naevi or scars) may also be included in the multidisciplinary approach of the identification process. The weighting attributed to each identifier is highly dependent on the nature of the disaster. Fire and putrefaction can alter DNA and fingerprints [5], and DNA persistence is limited by numerous factors such as time, type of tissue, and environmental conditions. Dental elements might also be lacking [2, 7, 8]. In the case of high fragmentation, genetic analysis may be the only way to re-associate fragmented elements together and to a common identity [2, 3, 9–11].

Another identification method is thus becoming more frequently used in DVI situations. In the case of commingled fragments, anthropological examination allows a preliminary re-association of skeletal elements to simplify consecutive works [10–13]. Anthropological age and sex estimation, combined with allometric relationships of long bones, are already used for minimum number of individuals estimation [14–16], and may also be useful as a preliminary step before re-associating commingled fragmented remains [2, 17].

Physical re-association (PRA) of fragmented bones allows fractured osseous elements to be re-assembled and fragments identified as belonging to the same bone element [18, 19]. This procedure is widely used by trained anthropologists, and was approved in 2018 by the American Academy of Forensic Science Board as a reliable method to re-associate fragments [18, 20]. However, this method may still require several days for cleaning and drying bones, and experienced specialists in the field. The physical reconstruction may be tedious, and some cleaning techniques may compromise subsequent DNA analysis [21, 22].

PRA of fractured elements is also a common method in archaeology, where it allows recreation of the initial morphology of fragmented artefacts, such as ceramics. Advances in 3D modelling and virtual re-association (VRA) have been used to prepare such reconstructions without risking further damage to the artefacts through extensive handling [23–28]. Furthermore, computer-aided virtual reconstruction of fragmented bones is now widely used for orthopaedic surgery. For this latter procedure, standard pre-operative computed tomography (CT)-scans and specific algorithms are being developed to automate the process [29–32].

Three observations can thus be made. First, CT imaging is now recommended in the main protocols of international standards [1, 5, 33]. Second, PRA of FHRs is a recognized method for associating fragments from the same bone, and thus from the same individual, although this method often demands a preliminary cleaning and drying process that can last several days and may alter the fragments themselves. Third, virtual methods for the reconstruction of fragmented objects are already used with satisfying results in archaeology and surgery; these VRA methods can be performed using standard CT imaging. During the DVI process, mobile or clinical CT scanners may thus be used to obtain virtual osseous models of the recovered remains, and then to virtually re-associate them, as is performed in pre-operative planning for orthopaedic surgery. In combination with the standard identification procedures, this method would decrease the quantity of DNA sampling and analysis, and would allow for quicker identification of fragmented remains. Moreover, a computer-aided method for re-associating FHRs may avoid the cleaning and drying procedure, and thus could be quickly initiated in parallel with the standard identification protocol, or even in a staggered way. Digital data are easier to store than biological items, which can quickly degrade and have to be preserved in negative temperature conditions. Finally, once the physical remains have been given back to the victims’ families, the virtual models can be preserved and used for forensic purposes, including trauma analysis.

Obviously, many factors need to be considered to adapt orthopaedic surgery or archaeological virtual reconstruction to this specific forensic identification context. In this respect, a previous literature review listed the existing computer-aided methods that could be used in a DVI situation, and resulted in the proposition of an innovative protocol [34].

The aim of this study was to evaluate the perspectives and limitations of such a VRA protocol, comparing it with the PRA method in a laboratory setting and a more realistic case scenario of a controlled bombing of a pig carcass.

## Material and methods

To answer our research questions, this study was organized into two different phases. In the first phase, reconstructions of fragmented bones were performed using VRA and PRA, and these were evaluated by comparing their accuracy, the time needed, and the percentage of re-associated fragments. In the second phase, the VRA was tested with fragments resulting from a controlled explosion using a porcine model, to evaluate the limits of the techniques in a more realistic context.

**Figure 1:**
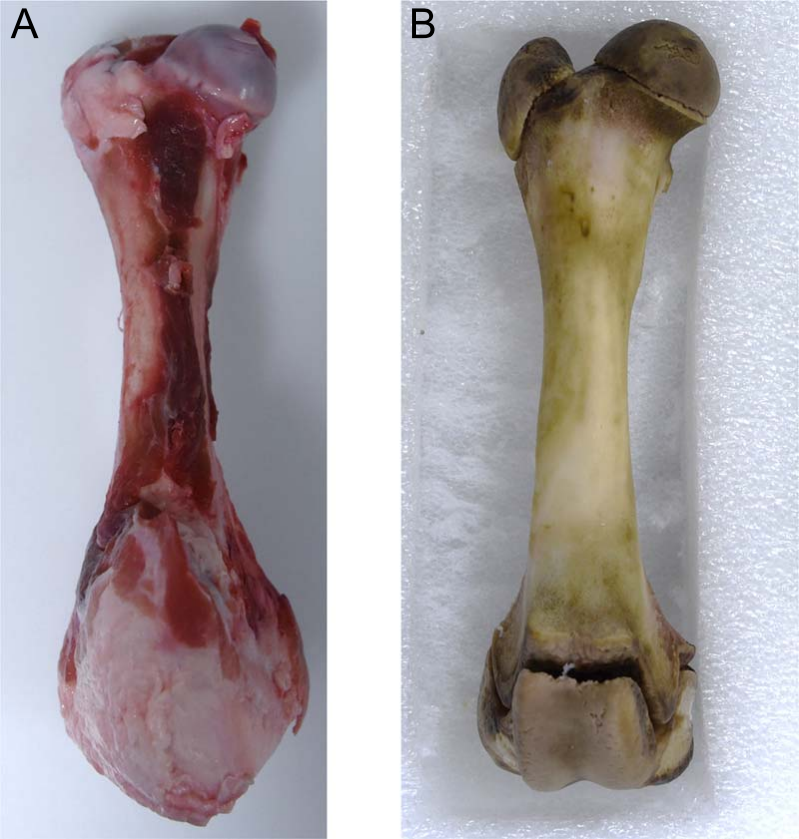
One of the six porcine femurs, before (A) and after (B) cleaning.

### Phase 1: laboratory setting

#### Cleaning and intact bone data acquisition

Six porcine femurs of immature pigs (*Sus scrofa domesticus*) obtained from the food industry were cleaned (Figure 1) using an enzymatic maceration bath prepared with 40 g of Enzyrim-Oss® forte (Bauer-Handels GMBH, Fehraltorf, Switzerland) and 40 g of Aniosafe® (Anios, Lezenne, France) diluted in 2 L of water. Femurs were first immersed for 24 h in a stove at a temperature of 60°C. Residual adherent tissues were removed using toothbrushes and scalpels, avoiding contact with the cortical bone. The femurs were then immersed in a renewed enzymatic maceration bath for 24 h at 60°C, as described previously. Cleaned femurs were then rinsed and dried on metallic grids for 24 h, and then on plastic trays for 7 d.

**Figure 3:**
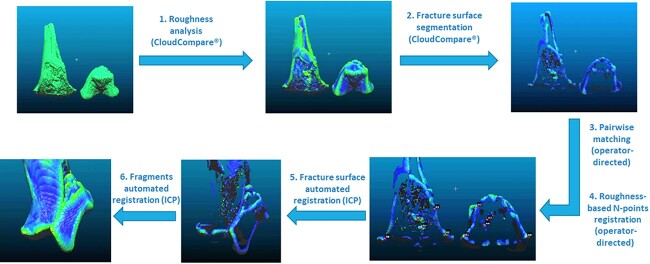
Description of the six steps of the virtual re-association protocol. ICP: iterative closest points.

Once dried, the six femurs were placed onto polyethylene foam to create a low radiodensity gap between the bone and the tray of the multi-detector computed tomography (MDCT) scanner. They were then scanned using a 64-row MDCT scanner (GE Light Speed VCT, Waukesha, WI, USA). A specific protocol for the acquisition was developed and applied to each case: 100 kV; 120 mA; pitch of 0.515; tube rotation of 1 s; 512 × 512 matrix; slice thickness/interval 0.625/0.315 mm, and reconstruction with a bone algorithm.

The resulting images in Digital Imaging and Communications in Medicine (DICOM) format were imported into Mimics® 24.0 software (Materialize®, Leuwen, Belgium), and were then segmented using region growing and seed placing techniques (“split” and “region grow” tools) to isolate continuous osseous tissue. The resulting 3D models were exported and saved as triangular meshes in standard tessellation language (STL) format.

#### Fragmentation of bones and fragmented bone data acquisition

**Figure 2:**
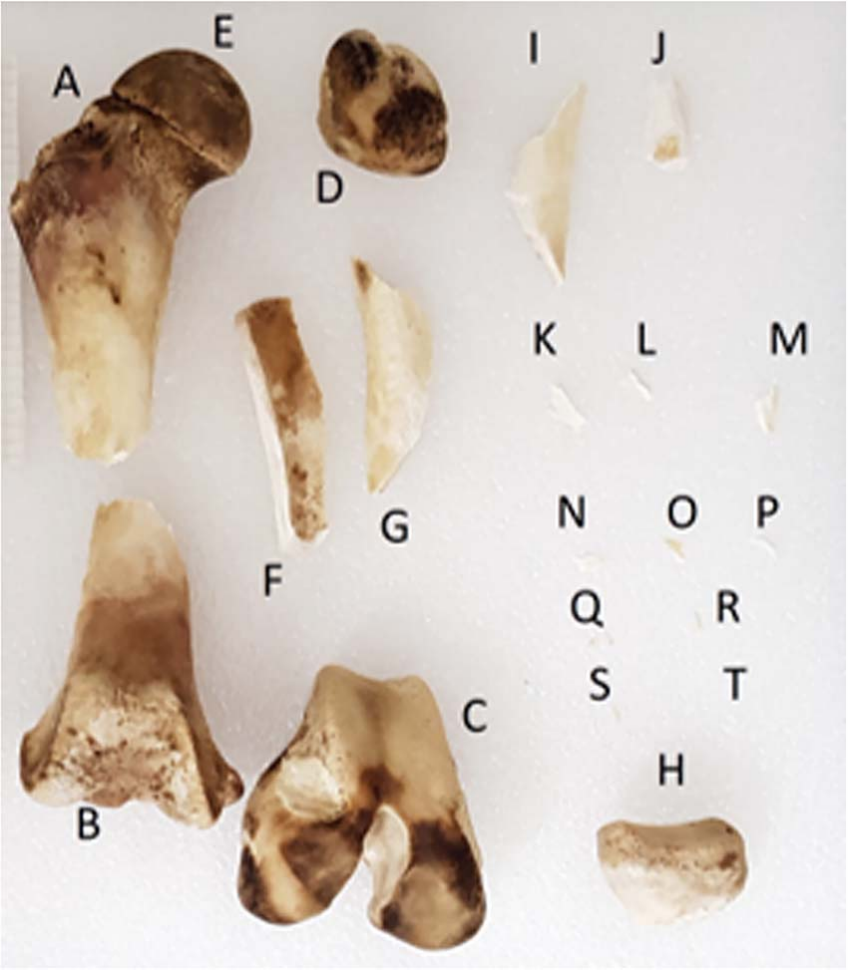
A porcine femur after manual fragmentation.

The six cleaned femurs were manually fragmented through multiple blunt force impacts delivered using a plain steel bar. Each set of fragments (Figure 2) was then scanned using the same 64-row MDCT scanner (GE CT750 HD) with the same protocol described above and image segmentation was performed with Mimics® software.

#### Virtual reconstruction

Reconstruction of the fragments was implemented in the freeware CloudCompare® v2.6.3 (https://www.danielgm.net/cc/), which allows for algorithm coding with the support of a wide dynamic digital community. The initial meshes were subsampled to 100 000-point clouds and the fragments were virtually re-associated and merged using the protocol developed in the initial critical review [34] (Figure 3):

1. The initial models were cloned, and a roughness analysis was performed on these secondary cloned models using the corresponding tool in the CloudCompare® freeware.
2. According to Irwansyah et al. [35], fractured cortical surfaces present higher roughness values than intact surfaces; therefore, a minimum threshold of 1.0 was set for the scalar roughness value, and points with lower roughness values were deleted. This operation resulted in the segmentation of fracture surfaces.
3. Matching surfaces were selected by the operator based on skeletal morphology and similarities of the surfaces. One point cloud was considered as the reference (fixed) model, and the other as the transformed (mobile) model.
4. Four points were selected on the reference and transformed point clouds to perform a preliminary registration using the iterative closest points (ICP) algorithm. This program adjusts the position of the transformed model towards the reference one, to reduce the distances between pairs of selected points to their lowest values.
5. The adjustment of matching fragments was refined by applying the ICP algorithm to the fractured point cloud, and the most distant points were excluded.
6. The transformation coordinates were copied and then applied to the initial fragments, to register the transformed fragment onto its corresponding fractured surface.

The point clouds of the reference and transformed fragments were merged to form a new model. This new reference fragment was then re-associated with the other fragments of the set.

#### Physical reconstruction and data acquisition of the final reconstructed bone

**Figure 4:**
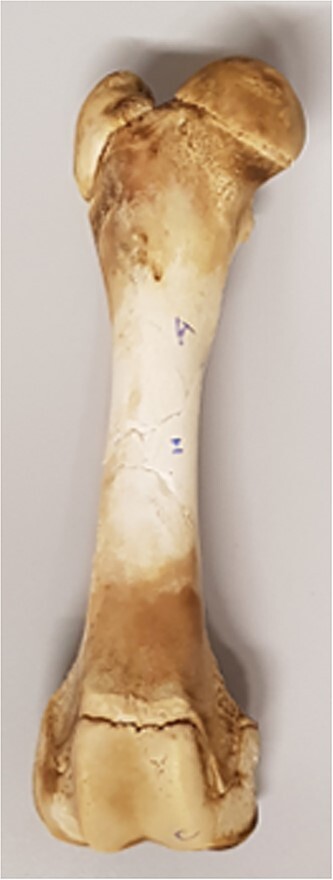
Full reconstruction of a porcine femur using the physical re-association method.

Using the different sets of fragments, the six femurs were physically reconstructed (Figure 4). A preliminary reconstruction was set up using tape, and then the pieces were glued using vinylic wood glue (Sader® quick drying; Bostik, Colombes, France). Wood glue presents the advantage that it can be removed with hot water, whereas cyanoacrylate glues require solvents for fragment reposition.

The resulting physical reconstructions (PR) were then scanned using the HD 750 MDCT scanner and the protocol described above. The DICOM files were segmented using Mimics® 24.0 software, and models were constructed using the protocol defined above.

#### Evaluation of physical and virtual reconstructions

**Figure 5:**
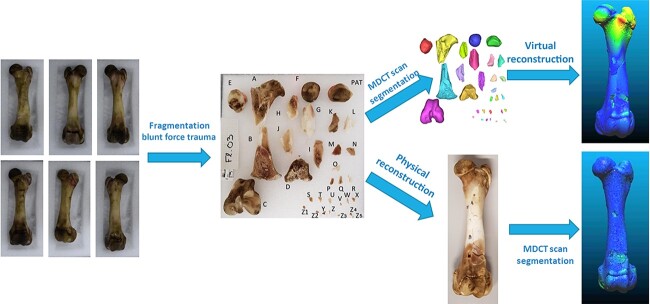
Applied methodology for comparing the virtual re-association (VRA) and physical re-association (PRA) methods. MDCT: multi-detector computed tomography.

The virtually and physically reconstructed models were registered onto the corresponding intact bone point cloud using an ICP algorithm, and the cloud-to-cloud distances were calculated with a root mean square algorithm. The mean distances (μVRA and μPRA) were saved as criteria for assessing the virtual and physical reconstruction accuracy (Figure 5). Both the time needed for the full virtual and physical reconstructions (tVRA and tPRA) and the number of re-associated fragments was also recorded.

Considering the small size of our sample set, and the simple comparison between the VRA and PRA series, the intraclass correlation coefficient was calculated with Excel for three criteria: the time needed for a full reconstruction, accuracy, and representativeness.

### Phase 2: controlled bombing

In March 2021, the Swiss police department in charge of legal operations involving explosive materials and the Swiss Human Institute of Forensic Taphonomy co-organized a controlled explosion using a porcine model as part of a training exercise for explosive specialists to evaluate the dispersion of fragmentation material in a simulated open-field bombing attack.

This training mission was used to additionally test the VRA approach in a “real case” scenario.

**Figure 6:**
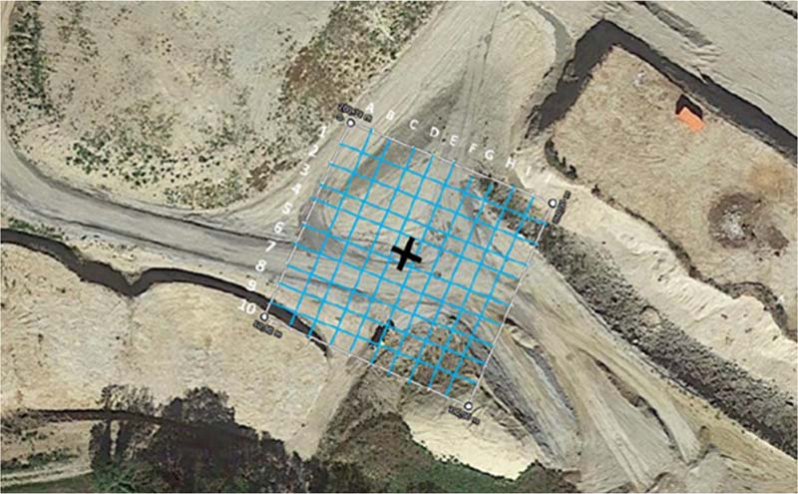
Initial gridding of the controlled bombing site.

A gravel pit was first gridded into 5 m × 5 m sectors on a surface of 50 m × 50 m on the basis of preliminary published results (Figure 6) [36].

The body of an adult pig *(S. scrofa domesticus)* was obtained from a local butcher and equipped with a vest in skin contact containing a total of 2 kg of Plastex explosive (Société Suisse des Explosifs SA, Brig, Switzerland) and 150 M8-size bolts as fragmentation material. The explosive was divided into 1 kg of plastex and 50 bolts placed in a ventral position and 1 kg of plastex and 100 bolts placed in a dorsal position. Plastex is a civil explosive used for quarry applications (dislocation of rocks) with a detonation speed of 7 300 m/s.

The body was hung on a gallows at the centre of the gravel pit and dressed.

The bomb was triggered from a distance and the projected biological fragments were then localized on the grid, individually bagged, and labelled. The CT protocol was modified from that used for the pig femurs to speed up the process of scanning: a real case scenario would need to scan a large amount of fragments in a minimum of data acquisition. The following CT parameters were used: 100 kV; 320 mA; pitch of 0.969; tube rotation of 1 s; matrix 512 × 512; slice thickness/interval (mm) of 0.125/1 for the primary reconstruction and 0.625/0.05 for a second reconstruction; reconstruction using a bone algorithm.

**Figure 7:**
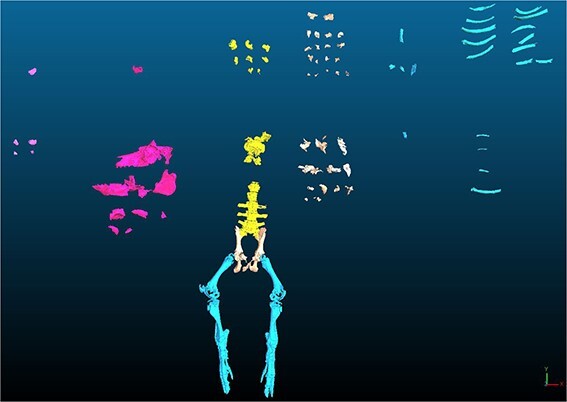
Three-dimensional models (STL files) of the fragments resulting from the bombing experiment, sorted in the CloudCompare® software.

The DICOM files were segmented with Mimics® 24.0 software, using region growing algorithm form seed placement: a pixel, or seed, is chosen inside the region to segment, and is considered as the reference for determining whether the neighbours pixels are considered as part of the object to segment or as excluded pixels. This process allows to dissociate fragments in contact. Osseous fragments of <2 cm long were excluded, and a preliminary triage was applied to prepare the matching fragments by type: the fragment models were sorted according to their bone type (diaphysis, flat bone, epiphysis-short bones), then by anatomical region (head, trunk, upper limb, lower limb) [13], and then, if possible, by bone (Figure 7).

## Results

### Phase 1: laboratory setting

**Figure 8:**
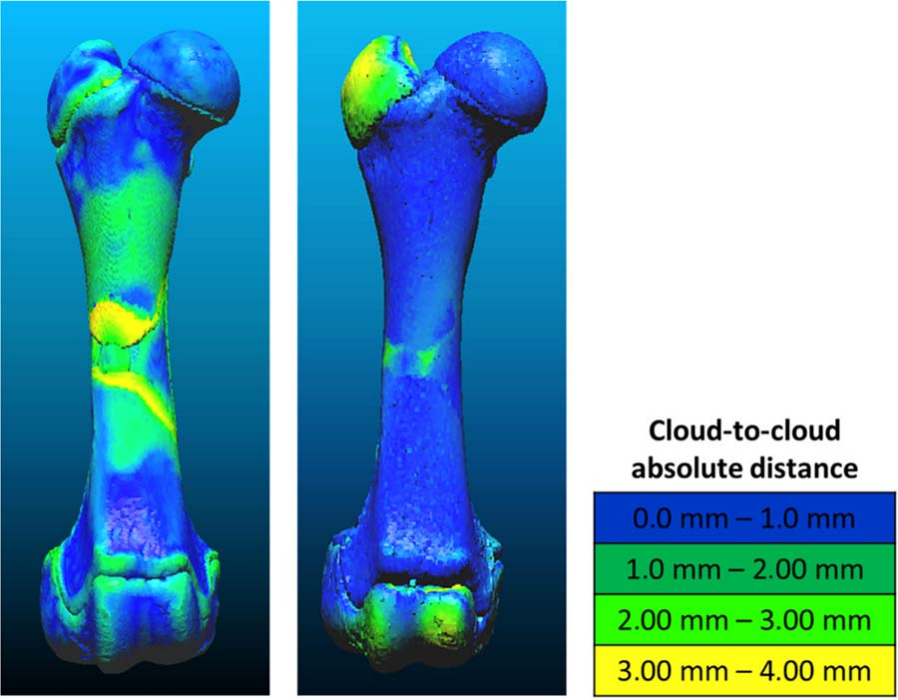
Colorimetric assessment of the cloud-to-cloud absolute distance between an intact bone and its full reconstruction using the virtual re-association (VRA) (left) and the physical re-association (PRA) (right) methods.

The manual fragmentation of the six porcine femurs resulted in a mean of 28.7 fragments per bone (standard deviation (SD) = 7.8; minimum = 20; maximum = 43) for a total of 172 fragments. Reconstructed models created using VRA and PRA methods (Figure 8 and Table 1) were compared using three criteria: the time needed for a full reconstruction, accuracy, and representativeness.

The mean time for the full virtual reconstructions (>95% of the bone surface reconstructed) was 131.8 min (SD = 20.9 min; minimum = 95 min; maximum = 150 min) per bone, which was 38% less time than that needed for the physical reconstructions (mean = 211.0 min; SD = 26.7 min; minimum = 177 min; maximum = 252 min).

The mean distance between the virtual reconstruction and the intact surface was 1.0 mm (SD = 0.3 mm; minimum = 0.5 mm; maximum = 1.3 mm), which is consistent with the mean distance of 1.1 mm obtained for the physical reconstruction (SD = 0.2 mm; minimum = 0.7 mm; maximum = 1.3 mm).

On the basis of this sample, the VRA presents results closely comparable with those of the PRA method.

The mean number of re-associated fragments was 12 (SD = 4; minimum = 8; maximum = 19) for the VR, and 16 (SD = 5; minimum = 9; maximum = 19) for the PRA. The virtual method reassociated fewer small fragments (maximum length inferior to 1.0 cm) than the physical reconstruction technique.

**Table 1:** Comparison of the results of the virtual re-association (VRA) and physical re-association (PRA) methods for the reconstruction time, the mean cloud-to-cloud distance, and the number of reassociated fragments.

Sets of fragmented femurs	Reconstruction time (min)		Mean distance (mm)		Number of re-associated fragments (*N*)		
	VRA	PRA	VRA	PRA	Number of fragments	VRA	PRA
F2.01	145	218	0.8	1.3	26	11	17
F2.02	120	177	0.5	0.7	20	8	9
F2.03	137	205	1.0	0.8	31	13	22
F2.04	150	224	1.2	1.2	26	14	18
F2.05	95	190	1.3	1.2	26	9	9
F2.06	144	252	1.3	1.2	43	19	19
Intraclass correlation coefficient	0.711514 20		0.614861 86		0.736882 58		

### Phase 2: controlled bombing

The outdoor bombing organized as professional training produced 983 biological fragments spread perpendicular to the bolt ejection cones. The hindlimbs were relatively intact without any femoral fractures, whereas the thoracic limbs were lacking, being either intensively smashed or blasted during the explosion. Massive fractures were observed on the costal ribs, vertebrae, coxal bones, and sacrum. These results are in accordance with existing findings [37]. Such an explosion in an open-field context leads to a high degree of fragmentation of the body elements that are in contact with the charge. After excluding fragments smaller than the minimal size criterion, a total of 87 fragments were included for the re-association (8.9% of the total material).

Among the 87 virtual models obtained after the MDCT acquisition of the collected fragments, only seven models (8%) matched based on their morphology and fracture edge profiles, resulting in three subsamples: the first one consisted of two cranial fragments, the second one of three mandibular fragments, and the last one of two costal fragments.

**Figure 9:**
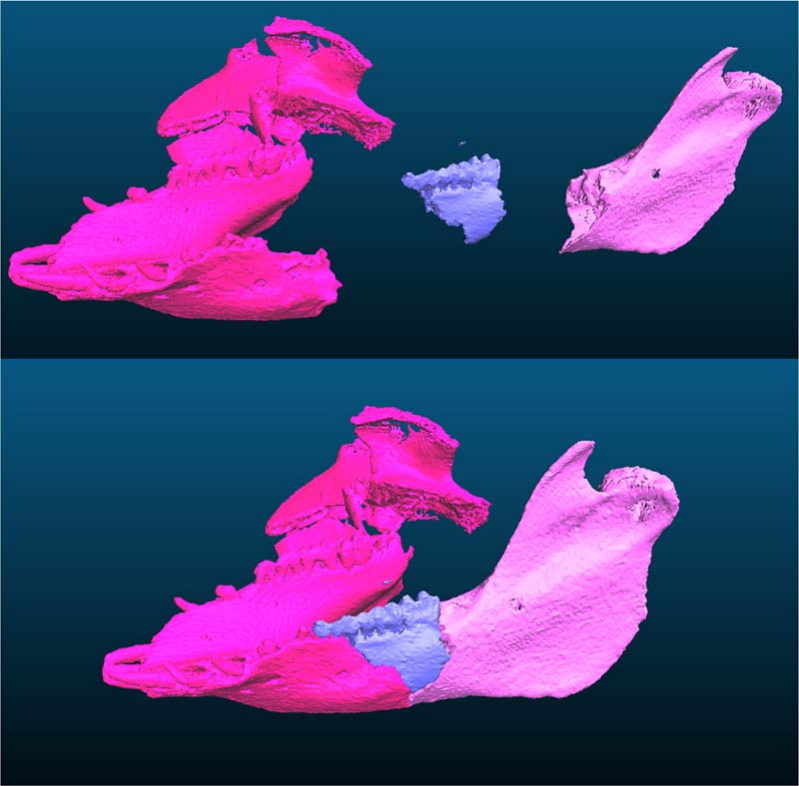
Virtual re-association (VRA) of three mandibular fragments.

The first steps of the VRA protocol were applied to matching fragments (Steps 1 to 4); however, the automatic refined registration did not lead to satisfying results, possibly because of a segmentation bias (Figure 9).

## Discussion

### DVI management and digital data

The management of a DVI mission is a complex process that has to be adapted to the wide variety of situations encountered [1, 5]: some strategical choices need to be made [2, 9, 38, 39] because of the limits of the standard identification method, the abilities of which can be overcome by a high degree of fragmentation or by alteration and commingling of the FHRs.

According to the present study, the acquisition of an MDCT scan may allow for a re-association of osseous fragments and a reduction in the number of genetic samples and analyses required. Radiographic analysis of bodybag contents is part of the international recommendation for a first triage of human remains and for searching for dangerous material and potential skeletal identifiers such as implants, healed fractures, or dental treatments [2, 4, 7, 10, 18, 33]. Thus, the possibility to acquire an MDCT scan and digital data seems to offer important value for the DVI mission. Once scanned, the fragments can be released for the standard process of identification.

However, access to an MDCT scan is not systematical, and depends on the existing medical structures and financial means of the country leading the DVI mission. Arming DVI teams with a mobile CT scanner should now be a prospect to be considered.

### Laboratory experiment

In the laboratory experiment, the influence of the operator’s experience of performing PRA on the reconstruction time was not evaluated. The reconstruction was performed using wood glue, which requires a long drying time (10 min for each reassembly) compared with other products such as cyanoacrylate.

Considering the VRA method, the original mesh was transformed into a 100 000-point cloud, whatever the size of the fragment. Subsampling point clouds of homogeneous density could allow for better standardization of the protocol, and should be evaluated, because it could possibly change the accuracy of the virtual reconstruction.

The preliminary results from our sample set of six porcine femurs showed no significant difference between the reconstruction accuracy of VRA and PRA, whereas the virtual reconstruction took 38% less time. Small fragments were excluded from the virtual reconstruction; however, they would not have been sampled for genetic analysis in a DVI situation. Moreover, this time calculation does not consider the cleaning and drying time needed to perform the physical reconstruction. Thus, the VRA method represents an important gain in time, and could be started immediately after the CT-scan, leading to matches before the DNA sampling process. It would also save the financial costs of sampling and genetic analyses for the considered fragments. In the case where a full reconstruction of the bone is required, the VRA method could be a useful tool for preliminary planning of the physical reconstruction.

However, the influence of fragment size on re-association efficiency also needs to be evaluated because it appears that small fragments (<1 cm) were uneasily integrated into the reconstruction. This may result from segmentation bias, and from the unconstrained registration algorithm that allowed interpenetration of the 3D surfaces.

Some methodological limits also need to be investigated: the sample of six fragmented femurs has to be expanded to evaluate the inter- and intra-operator variability of both reconstruction methods. Moreover, the porcine femurs were cleaned before fragmentation, whereas in a DVI situation, bones are embedded in muscles, tendons, ligaments, and cutaneous soft tissues, and the experimental conditions may create a bias for the type of fracture, and for the MDCT image segmentation. An evaluation of the method using fleshed bones is therefore needed. Additionally, the plastic deformation of fresh bones due to high pressure or high intensity impact (such as in explosions or air crashes) would probably alter the possibility of re-associating some fragments. To this end, a real case scenario involving a pig bombing was organized as a field experiment.

### Field experiment

The transposition of the VRA protocol developed for cleaned femurs fragmented by blunt force trauma to a full body fragmented with a controlled bombing showed important limits, with only 8% of the 87 considered fragments being re-associated. In this realistic situation of explosive fragmentation, this low matching rate may be explained by the loss of intermediate fragments due to the intensity of the explosion itself, to the ballistic effect of the metal bolts, and to the projection of fragments outside the collection area. Indeed, the segmentation was performed on fleshed bones with cartilage *in situ*. In addition, for multiple fragments, segmentation included the alveolar bone, and its irregular fractured surface may have compromised the accuracy of the registration. The presence of alveolar bone and dense cartilage in parts of some models made their reassociation difficult. The explosion also produced an important loss of intermediate bone elements, and the VRA parameters need to be investigated in order to assess the perspectives and limitations of the VRA technique in this fragmentation mechanism: the amount of explosive material and the site topography (such as the presence of reflection surfaces) are important parameters to be considered.

However, a positive effect from the simplification of the number of fragments can be noticed, even if VRA improvements appear mandatory. The field experiment allowed us to further consider the strategy for fragment collection, including small fragments with a minimum length of 1 cm, and the pertinence of a preliminary triage of the osseous models before VRA. This experiment allowed us to highlight the directions in which VRA must progress in order to assist in improving the efficiency of DVI records. Our VRA method may be faced with limits depending on the explosive charge and degree of fragmentation, but our results suggest that it can compete with and/or assist PRA when such an approach is required.

## Conclusion

While some methodological and technical limits have been highlighted, and further evaluations must be performed to validate the protocol for forensic anthropology use, our method developed for VRA of fragmented remains showed promising results in a controlled study. The preliminary tests allowed accurate reconstruction with the same efficiency as physical reconstruction, and an important gain considering the time required for reconstruction. This VRA method could be an efficient tool in DVI situations, in complement to the standard identification protocol, because it may be remotely performed.

Although there is a clear need for standardization of the on-site collection, and for the automation of the process to be used in high-fragmentation contexts, the VRA allows the matching of fragments of the same bone, and should avoid unnecessary genetic sampling and analyses, thus reducing the time and cost of the identification process.

This protocol could also have useful applications in the challenging context of commingled archaeological remains, mass grave situations, and the pre-operative planning of complex orthopaedic fracture reduction.
